# Preparation of Nanoparticle-Immobilized Gold Surfaces for the Reversible Conjugation of Neurotensin Peptide

**DOI:** 10.3390/biom15060767

**Published:** 2025-05-27

**Authors:** Hidayet Gok, Deniz Gol, Betul Zehra Temur, Nureddin Turkan, Ozge Can, Ceyhun Ekrem Kirimli, Gokcen Ozgun, Ozgul Gok

**Affiliations:** 1 Department of Nanoscience and Nanoengineering, School of Engineering and Natural Sciences, Istanbul Medeniyet University, 34720 Istanbul, Turkey; hidayetgok76@gmail.com; 2 Department of Biomedical Engineering, Institute of Natural and Applied Sciences, Acibadem Mehmet Ali Aydinlar University, 34752 Istanbul, Turkey; denizgol1997@gmail.com (D.G.); ozge.can@acibadem.edu.tr (O.C.); ceyhun.kirimli@acibadem.edu.tr (C.E.K.); 3 Department of Medical Biotechnology, Institute of Health Sciences, Acibadem Mehmet Ali Aydinlar University, 34752 Istanbul, Turkey; betulzkarakus@gmail.com; 4 Department of Physics Engineering, Faculty of Engineering and Natural Sciences, Istanbul Medeniyet University, 34720 Istanbul, Turkey; nureddin.turkan@medeniyet.edu.tr; 5 Department of Biomedical Engineering, Faculty of Engineering and Natural Sciences, Acibadem Mehmet Ali Aydinlar University, 34752 Istanbul, Turkey; 6 Department of Medical Biotechnology, Institute of Health Sciences, Acibadem University, 34752 Istanbul, Turkey; gokcen.ozgun@acibadem.edu.tr; 7 Department of Biomaterials, Institute of Natural and Applied Sciences, Acibadem Mehmet Ali Aydinlar University, 34752 Istanbul, Turkey

**Keywords:** reactive nanoparticles, poly(ethylene glycol), surface modification, coating, disulfide exchange reaction, neurotensin

## Abstract

Polymer coatings as thin films stand out as a commonly used strategy to modify biosensor surfaces for improving detection performance; however, nonspecific biomolecule interactions and the limited degree of ligand conjugation on the surface have necessitated the development of innovative methods for surface modification. To this end, methacrylated tethered telechelic polyethylene glycol (PEG-diMA) chains of three different molecular weights (2, 6, and 10 kDa) were synthesized herein and used for obtaining thiolated nanoparticles (NPs) upon adding excess amounts of a tetra-thiol crosslinker. Characterized according to their size, surface charge, morphology, and thiol amounts, these nanoparticles were immobilized on gold surfaces that mimicked gold-coated mass sensor platforms. The PEG-based nanoparticles, prepared especially by PEG6K-diMA polymers, were shown to result in the preparation of a monolayer and smooth coating of 80–120 nm thickness. Cysteine-modified NTS(8–13) peptide (RRPYIL) was conjugated to thiolated NP with reversible disulfide bonds and it was demonstrated that its cleavage with a reducing agent such as dithiothreitol (DTT) restores the NP-immobilized gold surface for at least two cycles. Together with its binding studies to NTSR2 antibodies, it was revealed that the peptide-conjugated NP-modified gold surface could be employed as a model for a reusable sensor surface for the detection of biomarkers of same or different types.

## 1. Introduction

Owing to their unique physical and chemical properties, nanoparticles (NPs) are considered powerful tools for use in medical fields ranging from drug delivery and regeneration to damaged tissue diagnosis and biomarker detection [1,2]. Due to their small size and high loading capacity, these nano-sized particles can be incorporated into several biomaterial platforms such as scaffolds or coated on medical device surfaces. Biosensor surfaces can be modified with functionalized NPs that contain abundant reactive groups and have a high surface-to-volume ratio; therefore, they can accurately detect disease biomarkers in biological fluids [3]. Reactive moieties on their periphery allow the conjugation of several active molecules, which enables more effective multivalent interactions with the biological counterparts in the body fluids, like serum, saliva, and sweat [4,5,6,7,8,9]. Additionally, biosensors must have high selective specificity and sensitivity to quantify target molecules. The limit of detection (LOD) for gold surface-based mass biosensors can be decreased by increasing their binding ability to target biomolecules in a mixture [10,11]. The multivalency provided by functionalized NPs on the biosensor surface can facilitate the rapid detection of small amounts of target molecules [12].

The properties of NPs can be enhanced for use in specific applications by leveraging the reactive groups on their periphery during surface modification [13,14]. Lipidic NPs, such as liposomes, and polymeric NPs with tunable physical properties have various peripheral functional groups and abundant binding sites; therefore, these are suitable for post-modification with biomolecules [15,16,17,18,19]. Metal NPs are coated with polymers to facilitate the binding of active biomolecules on their surface because the optical and electrical properties of metal NPs are coupled with the functionalization ability of polymeric coatings [20,21,22]. As NPs are functionalized with chemical reactive groups and used in the medical field, coating them with polymers is preferred for surface modification [23]. Therefore, NPs obtained from natural or synthetic polymers are considered ideal for improving the detection sensitivity and surface properties of biosensors [24,25,26]. The sensing properties of biosensors for reversible detection can be improved by modifying them with a stable coating of polymeric self-assembly layers or NPs. The obtained reusable biosensor surfaces can be used to detect specific materials such as bacteria [27,28].

Various biomolecules have been deposited or immobilized to gold surfaces in mass sensors such as quartz crystal microbalance (QCM) and surface plasmon resonance (SPR) sensors [29,30,31]. The detection sensitivity of biosensors can be enhanced by binding free thiol groups in biomolecules or polymers to their gold surfaces via semi-covalent bonds [32]. For instance, Triyana et al. coated the gold surface of a QCM sensor with thiol-modified chitosan—a natural polymer—via semi-covalent bonds [33]. Wu et al. modified the QCM sensor surface for protein detection by facilitating the binding of thiol-containing polymers on the gold sensor surface with nanosized protein-based receptors (IgG1); the modified sensor could detect the binding kinetics of cardiac myoglobin [34]. Yang et al. attached gold NPs on the gold surface of QCM sensors using an intermediate linker. Biomolecules attached to the NPs readily made complexes with boric acid and its derivatives; as a result, the sensor achieved high sensitivity and precise detection ability [35]. Diltemiz et al. coated a QCM sensor with polymeric fibers containing CuO–ZnO NPs for detecting formaldehyde (FA) at a low concentration (LOD) of 41 ppb [36]. The majority of studies have focused on integrating NPs into biosensors for the detection and quantitative analysis of heavy metals and gases in the environment and in industrial waste [37,38,39]. However, their applicability for biomarker detection in body fluids such as blood, urine, saliva, and cerebrospinal fluid to identify diseases has not been extensively explored yet [40,41,42,43].

Sensor surfaces modified with NPs have reduced LODs and improved detection accuracy; however, the interaction of unwanted compounds with ligands on the sensor surface has to be eliminated [44]. This issue can be addressed by increasing the surface roughness via hydrogen bonding and 3D conformation of biomolecules because nonspecific protein interactions prevent the binding of a target molecule to the surface. Bovine serum albumin (BSA) can be used for blocking the sensor surface and eliminating unwanted interactions with molecules [45,46,47]. However, BSA lacks covalent bonding and has low stability on the sensor surface. On the other hand, thicker BSA coatings can hinder target compound detection by reducing ligand availability [48,49]. Biosensor surfaces have also been recently coated with poly ethylene glycol (PEG) to inhibit such nonspecific protein interactions due to its anti-biofouling property and enhance their sensitivity [50,51]. PEG-coated surfaces were fabricated via physical coating or by chemically attaching polymer chains to the biosensor surface [52,53]. As PEG is highly hydrophilic, it forms a steric shell on the surface and prevents protein interactions. As a result, only the molecules with a high and specific affinity to the ligand on the biosensor surface are available for binding [54]. Mehne et al. reported that linear PEG polymers with different molecular weights could be integrated as a single layer onto the surface of optical sensors [55]. By increasing the length of linear PEG chains coated on the QCM surface, the adsorption of proteins such as fibrinogen, BSA, and lysozyme could be reduced. Moreover, high concentrations of PEG polymer solution led to reduced frequency shift and high dissipation in the QCM [56]. Branched PEG polymer coatings enhanced enzyme detection accuracy of a QCM; in particular, nonspecific protein binding decreased with increasing density of PEG on the sensor surface [57]. Electrochemical sensors coated with PEG-coated gold NPs could detect aflatoxin (an effective mycotoxin in milk) with high efficiency [58].

Polymer length impacts the detection of ligand molecules by reducing the ligand availability due to steric hindrance. Teramura et al. revealed that the molecular weights of PEG polymers coated on the gold surface of QCM sensors via lipid-based interactions modulated the degree of avidin–biotin complex formation [59]. A polymer with a molecular weight of 1 kDa could not prevent protein adsorption, whereas that with a molecular weight of 40 kDa had a low binding effect due to the steric (crowding) effect. The best result in this study was obtained using a polymer with a molecular weight of 5 kDa. This issue can be addressed using PEG polymers in different forms for surface modifications such as NPs. By not only introducing the PEG chains onto the surface, but also increasing surface area due to their spherical shapes, NPs have been evaluated as direct modification tools for improving the sensing ability of biosensors [60,61]. Boujday et al. investigated the effect of PEG coating on the assembly of Au NPs for the fabrication of a piezoelectric immunosensor to detect diclofenac, an anti-inflammatory drug. Compared to linear surfaces, they showed a significant enhancement in the biosensor sensitivity, up to six times the signal recorded for linear ones [62].

As PEG polymers can be functionalized with various reactive groups, they can be attached to the surface of sensors. The thiol-group-bearing linear PEG polymers can covalently link with the gold surface via semi-covalent bonding and facilitate stable surface coverage. However, this aspect has not been studied yet to the best of our knowledge. To address this gap, PEG-based NPs with free thiol groups were synthesized herein via radical-based cross-linking of methacrylated PEG polymers of different molecular weights under UV light. Their immobilization on gold-coated glass surfaces was investigated as a model platform for the gold surface of biosensors. Excess tetra-thiol-containing crosslinker was added into the reaction medium to obtain thiol-bearing NPs via thiol-ene chemistry. These functional groups were used for the immobilization of NPs onto gold-coated glass surfaces and the conjugation of a ligand, NTS(8–13) peptide; this ligand has a selective and high affinity to neurotensin receptor 2 (NTSR2) over-expressed in pancreatic cancer cell membranes. Peptide molecules were conjugated to the NPs via disulfide exchange reaction to provide peptide–target complexes on the NP-immobilized surface. These complexes could be washed with a reducing agent such as dithiothreitol (DTT) due to redox-responsive disulfide bond in between. The novelty of this approach is the reversible conjugation of a peptide to NPs and the availability of the NP-modified surface for another ligand attachment afterwards. The same surface can therefore be successively used for the conjugation of the same or a different type of ligands. The proposed strategy for obtaining a modular gold-coated surface is a straightforward yet powerful tool for developing reusable sensor surfaces.

## 2. Materials and Methods

### 2.1. Materials and Instrumentation

PEG polymers with different lengths (MWs 2, 6, and 10 kDa), methacryloyl chloride (MAC, 97%, MW 104.53 g/mol), Pentaerythritol tetrakis(3-mercaptopropionate) (>95%, MW 488.66 g/mol), Aldrithiol (98%, MW 220.31 g/mol), Irgacure2959 (98%, MW 340.32 g/mol), triethyl amine (Et_3_N, ≥99.5%, MW 101.19 g/mol), dichloromethane (DCM, anhydrous, ≥99.8%), and methanol (MeOH, absolute, ≥99.8%) were purchased from Sigma-Aldrich (St. Louis, MO, USA). DTT(>99%, MW 154.25 g/mol) was received from Biomatik Corporation (Cambridge, ON, Canada). Amicon^®^ Ultra-15 Centrifugal Filter Unit (Mwt cut-off: 100 kDa) was obtained from Merck Millipore (Darmstadt, Germany). N,N-Dimethylformamide (DMF for peptide synthesis, anhydrous, 99.8%), Fmoc-Cys(Trt)-OH (cysteine, MW 585.71 g/mol), Fmoc-Arg(Pbf)-OH (arginine, MW648.77 g/mol), triisopropylsilane (TIS, 98%, MW 158.36 g/mol), and oxyma pure (≥99.0%, MW 142.11 g/mol) were purchased from Sigma-Aldrich (St. Louis, MO, USA). Fmoc-L-Pro-OH (proline, MW 337.37 g/mol), Fmoc-L-Tyr(tBu)-OH (tyrosine, MW 459.53 g/mol), Fmoc-L-Ile-OH (isoleucine, MW 353.41 g/mol), Fmoc-L-Leu-OH (leucine, MW 353.41 g/mol), and rink amide proTide (resin, loading: 0.70 mmol/g) were obtained from CEM Corporation (Matthews, NC, USA). Piperidine (99%, MW 85.15 g/mol), N,N’-diisopropylcarbodiimide (DIC, ≥99.0%, MW 126.2 g/mol), diethyl ether (≥99.7%, MW 74.12 g/mol), and trifluoroacetic acid (TFA, MW 114.02 g/mol) were also acquired from Merck Millipore (Darmstadt, Germany). NTSR2 polyclonal antibody was purchased from ThermoFisher Scientific (0.5 mg/mL, PA1-41575) (Waltham, MA, USA). Acetonitrile (ACN, ≥99.9%) was purchased from ISOLAB Chemicals (Istanbul, Turkey). Syringe filters (0.22 and 0.45 μm, polyethersulfone (PES) and polyvinylidenedifluoride (PVDF) membranes) were sourced from TPP (Techno Plastic Products AG, Trasadingen, Switzerland). Phosphate-buffered saline (PBS) tablets were obtained from MP Biomedicals (Europe, Illkirch, France).

Peptide (CRRPYIL) was synthesized using a Liberty Blue™(CEM) automated microwave peptide synthesizer (Matthews, NC, USA). NPs were prepared under a UV lamp (365 nm, UVP, UVGL-58) and analyzed via dynamic light scattering (DLS, Wyatt Technologies DynaProNanoStar) (Santa Barbara, CA, USA) for their hydrodynamic volume with 10 successive measurements for each sample. A Malvern Zetasizer Nano ZS (Malvern Instruments Ltd., GB) was used for measuring the surface charge as zeta potential values (RT, *n*= 3). The purity of peptides and the antibody solutions were analyzed using an HPLC instrument (High-Performance Liquid Chromatography, ThermoScientific, DioNex Ultimate3000) (Waltham, MA, USA) that employed the C18 column as the stationary phase and %50 ACN and %50 ddH_2_O as the mobile phases with a flow rate of 0.1 mL/min. Fourier-transform infrared (FT-IR) spectra were obtained for the lyophilized samples via attenuated total reflectance (ATR) FT-IR spectroscopy (ThermoScientific NICOLET iS10) (Waltham, MA, USA) at 3500–500 cm^−1^. A Bruker 400 MHz (Bruker BioSpin, Rheinstetten, Germany) was used for ^1^H NMR spectroscopy in DMSO-*d*_6_. Gold-coated glass surfaces were fabricated in a vacuum sputter coater (Leica EM ACE200) (Leica Microsystems, Wetzla, Germany). A spin coater instrument (SCS, 6800 series) (Specialty Coating Systems, Indianapolis, IN) was used to fabricate polymer- and NP-coated surfaces. The morphology of NPsand coated surfaces was visualized via scanning electron microscopy (SEM-Quanta650) (Waltham, MA, USA) after coating with a 50 nm thick gold layer and using ETD under vacuum. SEM images were captured at 20 kV with a 3.5 spot size. Semi-quantitative elemental analysis was performed using an EDS detector connected to SEM. Contact angle measurements were performed using a Biolin Scientific AttensionThetaLite (Biolin Scientific, Stockholm, Sweden) (Optical Tensiometer with single autodispensing) against 5 µL dH_2_O. Peptide amounts on NPs were quantified using NanoDrop (Thermo Scientific™ NanoDrop™ One Microvolume UV–Vis Spectrophotometer, Waltham, MA, USA). UV–Vis spectra were acquired for Ellman’s assay (Shimadzu, UV-260) (Kyoto, Japan).

### 2.2. Synthesis of Methacrylated PEGs

PEG polymers with molecular weights of 2, 6, and 10 kDa with available primary hydroxyl groups at both ends were used. These polymers were first dissolved in toluene and subjected to azeotropic distillation twice in a rotary evaporator at ~50 °C, followed by incubation in a desiccator under vacuum overnight to remove moisture content. Then, these polymers (1.0 eq) were dissolved in anhydrous CH_2_Cl_2_, and MAC (2.2 eq) was added into the reaction media in a basic environment obtained using Et_3_N (1.0 eq). The reactions proceeded overnight, and the excess solvent was removed using a rotary evaporator under vacuum. The polymers were precipitated into cold diethyl ether and kept at −20 °C overnight for purification. The polymers were then filtered through a sintered glass (pore size 4) and dried in a desiccator. Based on the molecular weights of the collected polymers, yields were calculated as 99.9, 95.0, and 98.8% for PEG2K-diMA, PEG6K-diMA, and PEG10K-diMA, respectively. The addition of methacrylic ester into and the removal of hydroxyl groups from PEG polymers were verified via FT-IR spectroscopy.

### 2.3. Preparation of PEG-Based NPs

PEG-based NPs (NP1–3) were prepared viaradical-dependent cross-linking reactions with UV curing. A total of 27 mg of methacrylated PEG (PEG-diMA) polymer was dissolved in 8 mL of dH_2_O. Then, 10 µL of Irgacure (0.2% *w*/*v*) solution was added into this mixture as the photoinitiator, and the reaction continued for 20 min under UV light (365 nm) with vigorous stirring (at 1500 rpm). The as-obtained NPs were filtered through 0.22 and 0.45µm PES filters, and their hydrodynamic volumes were analyzed using DLS instruments (Wyatt Technologies DynaProNanoStar) (Santa Barbara, CA, USA). Their zeta potential values were measured using the Malvern DLS instrument after diluting with 0.01 M NaCl solution.

Across linker—pentaerythritol tetrakis (3-mercapto propionate)—was added to the reaction media to introduce free thiol groups (-SH) on the periphery of NPs, enabling thiol-ene chemistry for NP formation. During cross-linking, the polymer and photoinitiator dissolved in dH_2_O and crosslinker dissolved in absolute ethanol were added to each other as excess amount of thiol groups (1.5 eq to methacrylate groups on the polymer structure), so that free -SH groups were obtained in the final NP structure. The excess crosslinker was removed from the media via ultra-filtration by the centrifugation method using Amicon filters (MWCO 100 kDa) (Merck Millipore, Darmstadt, Germany). against ddH_2_O twice. The size and zeta potentials of the obtained NPs (NP4–6) were measured together with free thiol amounts that were determined using Ellman’s assay based on the manufacturer’s instructions [63].

### 2.4. Synthesis of Functionalized Peptide, (PDS-NTS(8–13))

Cysteine-modified peptide “CRRPYIL” (MW 919.14 g/mol) was synthesized by the Liberty Blue™ (CEM) (Matthews, NC, USA) automated microwave peptide synthesizer using a previously reported method; the synthesis scale was adjusted to 0.1 mmol resin and the C-terminus of the peptide sequence was modified to an amide end [64]. In brief, the resin (0.143 mg) was weighed and allowed to swell for ~30 min before peptide synthesis. DIC and oxyma were used as an activator and activator base, respectively, and piperidine was used for deprotection. Required amounts of amino acids were dissolved in an appropriate volume of DMF, as determined by the peptide synthesizer. The reaction was continued for ~1.5 h, and the crude peptide was cleaved from the resin using the razor peptide cleavage system (CEM) with a cleavage cocktail containing TFA (4.75 mL), TIS (125 μL), and ddH_2_O (125 μL) at 38 °C for 30 min. The cleaved peptide was then precipitated in 15 mL of cold diethyl ether and centrifuged twice at 8000 rpm for 5 min. Then, diethyl ether was decantated, and the peptide was dried in a desiccator overnight (yield: 66.4%, purity: 96.7% determined by HPLC). The chemical structure of the resulting pure peptide was characterized using proton NMR (in DMSO-*d*_6_) and FT-IR spectroscopies.

The thiol group of peptide C-NTS(8–13) was activated by the pyridyl disulfide (PDS) moiety to conjugate it to the periphery of thiolated NPs via the disulfide exchange reaction. The peptide (50.0 mg, 0.055 mmol) was dissolved in 2 mL of absolute methanol, and aldrithiol (17.9 mg, 0.081 mmol) was added to this solution. This mixture was stirred vigorously at room temperature overnight and in the dark. After the completion of the reaction, the crude was added to 100 mL of cold diethyl ether and the product was precipitated overnight at −20 °C. Pure PDS-Pep compound (32.8 mg, MW 1028.3 g/mol) was collected at the bottom of a falcon tube via centrifugation and dried in a desiccator (yield: 59.1%, purity: 80.8% determined by HPLC). The chemical structure of the compound was verified using proton NMR (in DMSO-*d*_6_) and FT-IR spectroscopies.

### 2.5. Preparation of Peptide-Conjugated NPs (Pep-NP)

Based on the amounts of free thiol groups, NPs prepared using PEG2K-diMA (NP4) and PEG6K-diMA (NP5) were chosen for peptide conjugation via the disulfide exchange reaction. Based on the thiol amounts on NPs, peptide molecules were used 1.5 eq excess to demonstrate their full conjugation to the NP surface. A total of 2.3 mg (2.223 mM) and 2.7 mg (2.656 mM) of PDS-activated peptides were used for conjugating to NP4 bearing 1.482 mM -SH and NP5 bearing 1.771 mM -SH in their structure, respectively. Briefly, PDS-Pep molecules were dissolved in 1 mL MeOH, and after their dissolution, 1 mL of the NP solution was added dropwise. The obtained reaction mixtures were stirred at 100 rpm overnight and at room temperature in the dark. The peptide-conjugated NPs were purified from excess peptide molecules and side products such as prydine 2-thione via ultrafiltration by centrifugation using Amicon filters (MWCO 100 kDa) against ddH_2_O twice at 3000 rpm for 5 min. The obtained NPs (NP7 and NP8, respectively) were analyzed for their size, zeta potential, and peptide amounts in their structures using NanoDrop (ThermoScientific, Waltham, MA, USA).

### 2.6. Surface Coating with NPs

The as-prepared NPs with and without thiol groups were coated on bare glass and gold-coated glass surfaces to simulate the surface of a gold-based sensor. NP solutions were stirred in an orbital shaker for 10 min at room temperature to obtain well-dispersed particles. Linear PEG polymers (with MWs of 2, 6, and 10 kDa) were prepared at a concentration of 27 mg/mL, i.e., the one used for synthesizing NPs. Subsequently, glass coverslips were dipped into these solutions for 30 min and shaken at 500 rpm, and these surfaces were placed into a spin coater instrument and subjected to vacuum for 1 min at 1000 rpm to completely coat the surface. The wettability of these surfaces was analyzed by measuring the water contact angle using the Biolin Scientific AttensionThetaLite device (Biolin Scientific, Stockholm, Sweden). The water contact angle was determined using the sessile drop method, wherein 5 µL of distilled water was dropped onto the prepared coated coverslips manually using Hamilton needles. An imaging system captured dozens of images of the water droplet in a very short time.

Glass coverslip surfaces were coated with gold using a vacuum sputter coater (Leica EM ACE200) (Leica Microsystems, Wetzla, Germany) and thoroughly cleaned in an ultrasonic bath in acetone, ethanol, and distilled water, sequentially; then, they were dried under nitrogen gas. An approximately 100 nm thick gold layer was coated on the surface of these cleaned glasses using the metal coating device, and the obtained gold-coated surfaces were stored under inert conditions. NP solutions were coated on these freshly prepared gold-coated surfaces using the aforementioned method.

The morphology of NP- and linear-polymer-coated glass and gold surfaces was evaluated via SEM. All samples were covered with a 50 nm thick gold coating to ensure high conductivity and chemical stability. The semi-quantitative analysis of sulfur was performed using the EDS detector to confirm the presence of NPs on the gold surface. To measure the thickness of the coatings deposited on the surfaces, they were cut into small sections with a glass cutter and placed vertically on the stub of an SEM. After coating the cut-edge with a ~50 nm thick gold layer, the cross-section thickness of the vertically placed glass was examined under a microscope in a tilted position.

### 2.7. Reversible Peptide Immobilization

Gold-coated glass surfaces were modified first with thiolated NPs. Then, the reversible attachment of peptide NST(8–13) was investigated by successive disulfide bond formation and cleavage using a strong reducing agent, DTT [64]. Separate surfaces were coated with NP5 (prepared using PEG6K-diMA) using the method described in Section 2.6. The PDS-modified peptide was dissolved in 50% MeOH and 50% ddH_2_O (*v*/*v*) until a final concentration of 0.5 mg/mL was achieved. The NP-coated surfaces were dipped in the peptide solution and incubated for 3 min in an orbital shaker at 200 rpm and room temperature. After rinsing the surfaces with ddH_2_O twice, they were subjected to a 50 mM DTT solution for 30 min at room temperature. These treatments with water washes in between were repeated twice. After each step, one surface was reserved and air-dried for further morphological analysis via SEM with an EDS detector in the mapping mode.

### 2.8. Binding Experiments

#### 2.8.1. Affinity Study

An RED-NHS 2nd generation protein labeling kit and Monolith NT.115 premium capillaries were obtained from NanoTemper Technologies (Munich, Germany). The binding affinity of the NTSR2 antibody–NTS(8–13) peptide interaction was evaluated using microscale thermophoresis (MST) with Monolith NT.115 Pico instrument (NanoTemper Technologies, Munich, Germany). The NTSR2 antibody was labeled with the Monolith protein labeling kit and RED-NHS 2nd generation kit and used as a target molecule in PBS with 0.005% Tween-20. Experiments were conducted using the Monolith NT.115 premium capillariesat Pico-RED 80% excitation power and medium MST power with thermophoresis. For the labeled NTSR2 antibody, a concentration of 20 nM was used, whereas for the NTS(8–13) peptide, 16 serial dilutions were prepared starting from 0.5 mg/mL. Dark incubation was performed for 20 min after mixing the target and ligand molecule [65]. Triplicate results were analyzed using the M.O. affinity analysisv.2.3 software.

#### 2.8.2. Surface Attachment/Detachment Study

Gold surfaces were first coated with thiolated NP6 (Section 2.6) and then conjugated with PDS–peptide molecules (Section 2.7). NTSR2 antibody solution was prepared by diluting 110 µL solution (containing 55 µg of antibody) into 190 µL freshly prepared and filtered 10 mMpH7.4 PBS buffer. The obtained antibody solution with a final concentration of 0.0034 nM was first analyzed using the HPLC instrument over a C18 column with a mixture of 50%ACN and 50% ddH_2_O (*v*/*v*) with a flow rate of 0.1 mL/min, and then used to incubate the air-dried surface. At different time points, a sample of 5 µL was withdrawn for HPLC analysis using the autosampler of the instrument. As such, continuous incubation was monitored up to 8 h. The removed surface was then dipped in pH3.0 acetic acid solution for 2 h and subjected to HPLC analysis to determine amount of antibody detached from the surface.

## 3. Results and Discussion

### 3.1. Preparation of PEG-Based NPs

**Figure 1:**
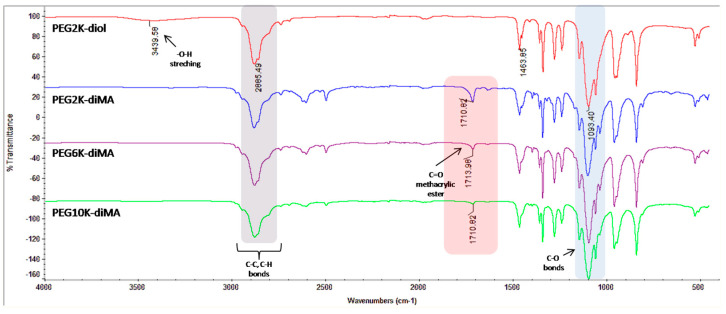
FTIR spectra of the synthesized PEG-diMA polymers and PEG2K-diol.

NPs were synthesized via the chemical cross-linking of methacrylate groups introduced to the ends of linear PEG chains. PEG polymers with molecular weights of 2, 6, and 10 kDa were used for synthesizing NPs with optimum properties such as size and surface charge. The reaction of primary hydroxyl groups with the methacryloyl chloride under the basic environment afforded the addition of methacrylate groups to both ends of the polymer chain; this modification was evaluated by FT-IR spectroscopy. A comparison of the FT-IR spectra for the polymers with that of a linear PEG2K showed that the stretching vibration peaks of primary hydroxyl (–OH) groups disappeared, which are normally observed as broad peaks at ~3400 cm^−1^. Figure 1 shows the appearance of new carbonyl bonds (–C=O) in the methacrylate groups at ~1710 cm^−1^ for the synthesized polymers with different molecular weights.

**Figure 2:**
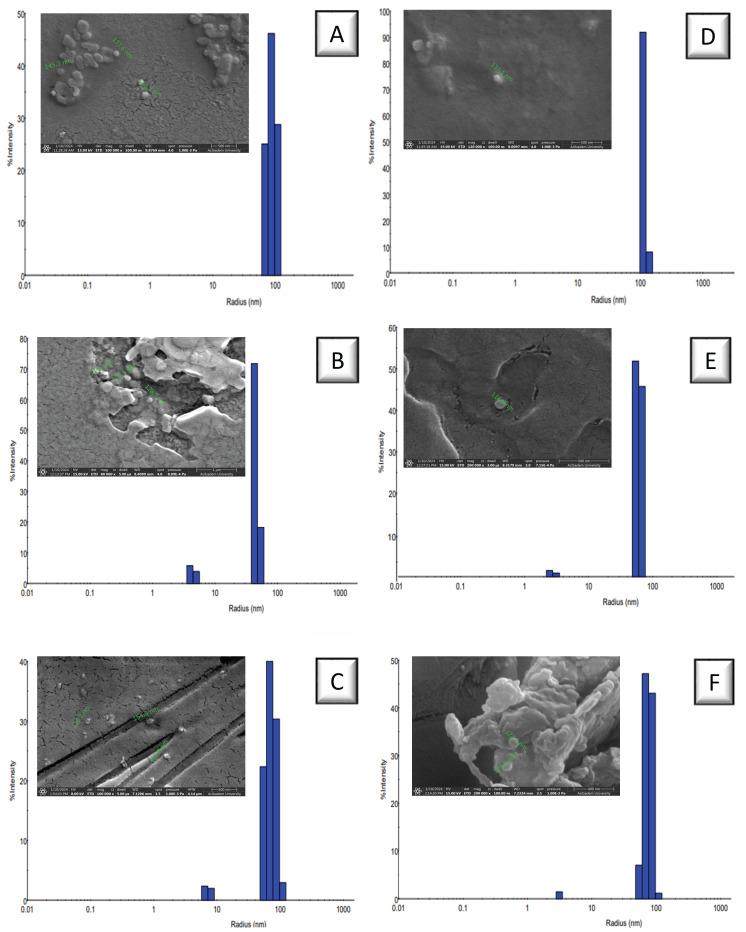
DLS results in aqueous environment and SEM images of nonthiolated ((**A**–**C**) as NP1, NP2, NP3) and thiolated ((**D**–**F**) as NP4, NP5, NP6) NPs. (Scale bar: 500 nm).

The PEG-diMA polymers (with molecular weights of 2, 6, and 10kDA) were cross-linked under UV light after the radical generation of Irgacure to yield PEG-based NPs (NP1, 2, and 3, respectively). Free thiol groups were introduced into the NP structures via an excessively used tetra-thiol-containing crosslinker via thiol-ene chemistry to obtain thiolated NPs (NP4, 5, and 6, respectively). The aggregates were removed by filtering NP solutions through 0.22 µm PES and PVDF syringe filters successively, and impurities with low molecular weights were separated via ultra-filtration by centrifugation using Amicon filters (MWCO100 kDa). All NPs were characterized for their hydrodynamic volume, zeta potential, and morphology. Table 1 shows the characterization parameters for all NPs; NPs prepared using PEG2K-diMA had the largest size, probably due to enhanced interaction of methacrylate units at both ends of the polymer chains of smallest length. The high intensity and low polydispersity index revealed that all NPs were almost uniform and homogeneous (Figure 2). Their size was measured as smaller under SEM compared to DLS measurement due to high vacuum conditions for visualization under microscope (Figure S2). Ellman’s assay was applied to NP4–6 to measure the free thiol amount in their structure for further conjugation. NP5 obtained using PEG6K-diMA contained the highest amount of thiol groups. The presence of sulfur atoms in the structure of the NPs was also verified via semi-quantitative elemental analysis performed via EDS combined with SEM (Table S1). All thiolated NPs preserved more negative surface charges compared with nonthiolated NPs. Although it is correlated with the thiol amounts obtained from Ellman’sassay for NP4 and NP5, the largest NP obtained from PEG10K-diMA (NP6) may have possibly induced the lowest surface charge.

### 3.2. Coating of NPs on Gold Surfaces

Gold-coated glass surfaces that served as a model for sensor surfaces were prepared in a vacuum sputter coater. The surface modifications using NPs were investigated to analyze their interaction with nonthiolated and thiolated NPs. The surfaces covered with a 100 nm thick gold film were coated with NP solutions using a spin coater instrument and air-dried. Their wettability and surface morphology were then analyzed. Table 2 shows that, compared with a bare gold-coated glass surface, PEG-based NPs provided the NP-coated surfaces with high hydrophilicity similar to linear PEG polymers (Table S2).

**Figure 3:**
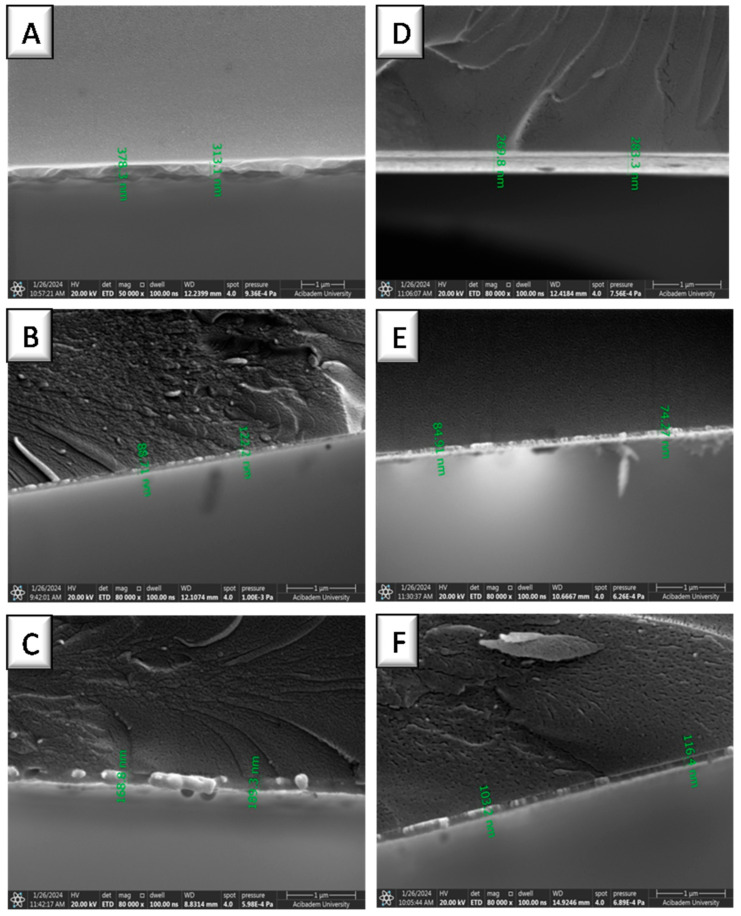
SEM images of the cross-sections of nonthiolated ((**A**–**C**) as NP1, NP2, NP3) and thiolated ((**D**–**F**) as NP4, NP5, NP6) NP-coated gold surfaces. The images were taken in tilted position.

NPs coated on gold surfaces were visualized via SEM, where spherical structures can be observed as distributed all over the surface. Moreover, the NP-coated gold surfaces were carefully broken into two with the help of a tweezer, and the thickness of the NP layer on the gold surface was measured by visualizing the surfaces from a tilted angle. Figure 3 presents the NP layers on gold-coated surfaces as thin films, where NPs prepared by PEG2K-diMA polymer (for both thiolated and nonthiolated ones) resulted in thicker coatings with multilayer NP deposition. On the other hand, monolayer NP distribution was observed from the side-view of coatings prepared using the same coating method. The smallest hydrodynamic volume was measured via DLS for NP2 and 5 prepared using PEG6K-diMA polymer; therefore, they formed thinner NP monolayers on the gold surface. However, NP5 formed a more uniform and homogeneous coating due to the formation of semi-covalent bond between the gold surface and its thiol moieties. To confirm this attachment, NP5 was incubated in ddH_2_O for 3 h with gentle shaking and EDS analysis was performed. SEM revealed the presence of NP layer on the gold surface, and elemental analysis confirmed the presence of sulfur atoms on the surface, which indicated the immobilization of thiolated NPs on the gold-coated surface [66] (Figure S4).

### 3.3. Peptide-Modified NPs

The conjugation of biomolecules to NP-decorated gold surfaces was achieved via the chemical binding of NTS(8–13) peptide to the thiol groups on the gold surface. NTS(8–13) is a fragment of the neurotensin peptide that has a specific and high affinity to neurotensin receptors (NTSR) over-expressed in cancer tissues [67]. NTS(8–13) peptide has a specific affinity to NTSR2, which is abundantly found in pancreatic cancer cell membranes [68]. For a comparison, the binding of this peptide to the NTSR2 antibody was studied against NP-coated gold surfaces as a model for the sensor surface. After the immobilization of NPs on the gold surface, the left-over thiol groups on NPs were evaluated for the conjugation of NTS(8–13) peptide via disulfide bonds. As a result, a biomolecule complex of peptide and antibody would be formed on the surface. This surface could be restored by treating with a reducing agent upon the cleavage of the disulfide bond between nanoparticles and peptide molecules.

**Figure 4:**
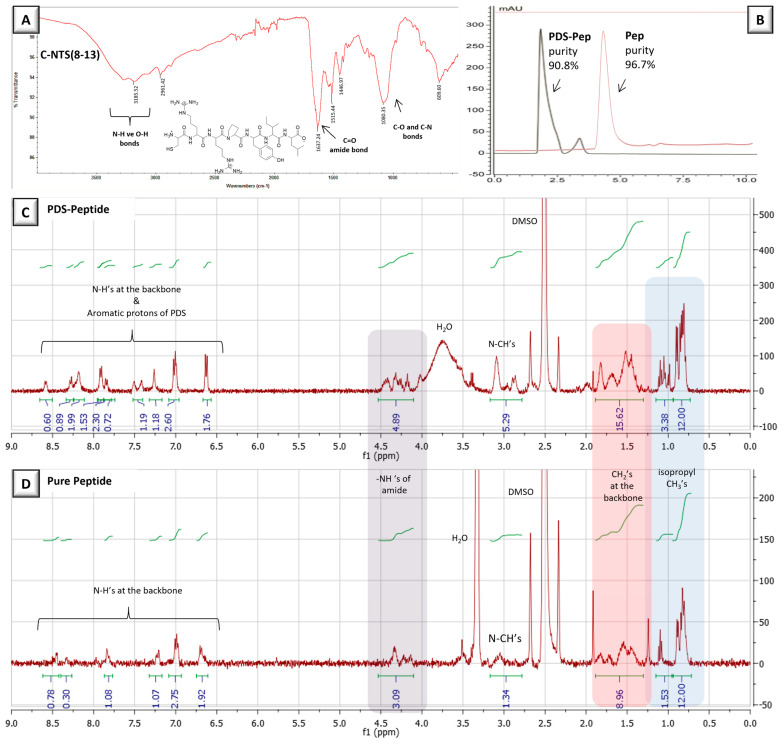
(**A**) FT-IR spectrum of Pep (NTS(8–13)), (**B**) HPLC chromatograms of Pep and PDS-Pep, (**C**) ^1^HNMR spectrum of PDS-Pep, and (**D**) ^1^HNMR spectrum of Pep (taken in DMSO-*d*_6_).

NTS(8–13) peptide was synthesized with an additional C (cysteine) residue at the N-terminal via solid-phase peptide synthesis using the peptide synthesis instrument. An amine resin was used for the growth of the peptide sequence; after cleavage, peptide molecules were purified via precipitation in cold ether. FT-IR spectrum of the peptide confirmed its chemical structure based on the characteristic peptide peaks observed at ~3200 cm^−1^ corresponding to N-H and O-H stretching along with a peak corresponding to an amide carbonyl peak at 1637 cm^−1^ (Figure 4A). The peptide had 96.7% purity, as revealed via HPLC analysis (Figure 4B). The thiol group from the cysteine amino acid was protected with the PDS group by the reaction of aldrithiol, with a yield of 59.1% and 80.6% purity. The PDS group attached to the peptide was verified via the peaks observed between 6.5 and 8.5 ppm in the ^1^HNMR spectrum (Figure 4C,D). The NMR spectra of pure peptide and PDS-modified peptide presented peaks related to the peptide structure at ~4.3 ppm for amide carbonyl protons and ~0.8 ppm for isopropyl –CH_3_ protons. This peptide was bound to the remaining thiol groups found on the NPs that were previously coated on the gold surface.

**Figure 5:**
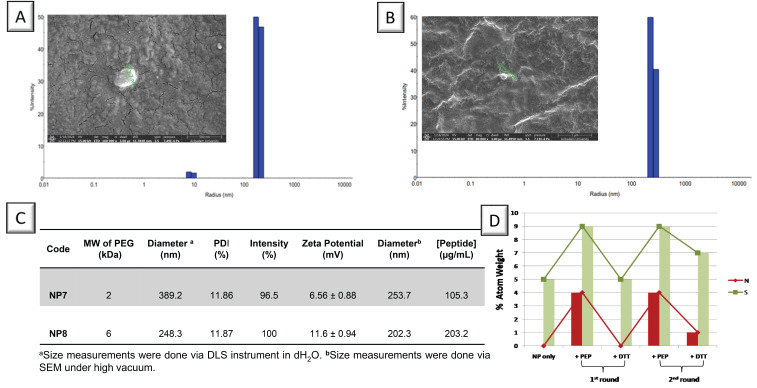
(**A**,**B**) DLS results and SEM images for NP7 and NP8, (**C**) characterization details, and (**D**) atomic percentages for NP8-coated surface via SEM-EDS mapping.

Peptide molecules were conjugated to the periphery of PEG-based thiolated NPs via disulfide exchange reaction. After Pep-NPs were purified using Amicon filters to remove any impurities and unreacted molecules, peptide attachment was verified by their increased size determined via DLS and SEM (Figure 5A,B). The NPs had also positive surface charges, indicating the successful attachment of peptide molecules. The peptide concentrations were measured using NanoDrop, using a calibration curve obtained based on pure peptide standards, resulting in 105.3 µg/mL peptide for NP7 (Pep-PEG2K NP) and 203.2 µg/mL peptide for NP8 (Pep-PEG6K NP) (Figure 5C). Ellman’s assay was applied to peptide-attached NPs to confirm that no free thiol groups were left after conjugation. As NP8 contained a high peptide amount, it was chosen for the following surface binding experiments.

To prepare NP-immobilized gold surfaces that could be reused for the binding of ligand molecules, PDS-activated peptides were attached to these surfaces via successive peptide binding and detachment steps (Figure 5D). An NP8-coated gold surface was modified by two successive cycles of peptide conjugation and cleavage via a reducing agent, DTT. EDS mapping and SEM imaging revealed that the atomic percentages of N and S increased after peptide conjugation and restored back to the condition of only NP-immobilized gold surface after DTT treatments. This behavior was observed for at least two cycles, indicating that the peptide could be completely removed by cleaving the disulfide bond via DTT. It can be concluded that the NP-coated gold surface could be restored back and used for attaching other peptide molecules of either the same or different types.

**Figure 6:**
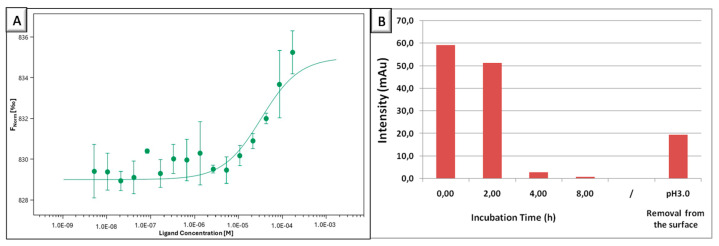
(**A**) Binding affinity of NTS(8–13) peptide for NTSR2 antibody via microscale thermophoresis (*n* = 3), (**B**) changes in antibody amount upon incubation over peptide-conjugated NP-coated gold surface over time and its release in acidic environment.

The binding ability of NTS(8–13) peptide was evaluated against the NTSR2antibody both in solution in the free form and on the surface as attached to NPs. Several dilutions of peptide were prepared and analyzed against RED-NHS-labeled antibody in the Monolith NT.115Pico instrument. Figure 6A shows the binding curve of the NTS(8–13) peptide–NTSR2 antibody interaction presented as FNorm[‰]—ligand concentration as molarity [M]. The experimental dissociation constant (Kd) of this interaction was calculated to be 3.29 ± 0.69× 10^−5^ M (32.9 ± 0.69 µM), indicating a certain level of affinity between the peptide and the antibody. According to the literature, the binding affinity of this NTS(8–13) peptide to its high-affinity receptor (NTSR2) was reported as a Kd value of 5–7 nm; [69] indicates a stronger interaction compared to that found in this study. Although this interaction was studied in solution, peptide multivalency provided by NPs on the surface was shown to help bring this binding tendency to higher antibody intensity. After the thiolated NPs (NP6) were coated on the gold surface, the thiol moieties on the surface were conjugated to PDS-modified NTS(8–13) peptide molecules and peptide-conjugated NP-coated gold surfaces were formed. This surface was incubated in an NTSR2-antibody solution of 3.4 pM, during which samples were withdrawn from the antibody solution and the decrease in the free antibody amount was determined via HPLC analysis (Figure S5). The amount of antibody used in this experiment was lower compared to its Kd value against this peptide to eliminate the saturation condition during the binding process, but still it is detectable in liquid chromatography. Figure 6B shows the measured antibody intensity in the incubation solution. Results revealed that 4 h of incubation was sufficient for the binding of ~95.2% of the antibody to peptides on the NPs. Moreover, the bound antibody was released under harsh acidic conditions (pH3.0), resulting in the detachment of 34.5% of the initially bound antibody amount on the gold surface. The HPLC chromatogram shows an extra peak in the nonpolar region, possibly indicating the deprotonation or denaturation of some of the released antibody (Figure S6). These findings indicated that the peptide-decorated NP-coated gold surface has the potential to be utilized as a reusable platform for binding studies.

## 4. Conclusions

Herein, a new strategy for preparing nanoparticle-immobilized surfaces was introduced that can be applied to gold-surface-dependent mass sensors to improve biomolecule detection. PEG-based polymers of three molecular weights (2, 6, and 10 kDa) were methacrylated from both ends with high yields of up to 95% and used for synthesizing NPs. These NPs were decorated with free thiol groups by adding excess amounts of a tetra-thiol-containing crosslinker. Thiolated NPs of 100–200 nm preserved negative surface charges and offered stabile hydrodynamic size in an aqueous environment. These NPs were coated on gold films deposited over glass surfaces, and thiolated NPs became immobilized via semi-covalent S–Au bonds to these surfaces. The side-view of NP-coated gold surfaces revealed the thickness of nanoparticle coatings to be ~250 nm. The thickness notably decreased and a monolayer of NPs was formed on the gold surface, particularly with those synthesized fromPEG6K-diMA polymers, that had a size of 85 nm. The thiol groups on NPs were attached to a peptide sequence (NTS(8–13)), obtained with a purity of 95%, which is known for its high affinity to receptors in pancreatic cancer cells. Its reversible conjugation via disulfide bonds facilitated its release after biomolecule binding, and the NP-immobilized surface was shown to be restored for subsequent use for at least two cycles. The binding of this peptide to its receptor antibody was investigated to reach up to 96.7% within 4 h. These findings indicate that the developed protocol for the preparation of an NP-immobilized gold-coated glass surface can be evaluated as the one to be applied to gold-film-involved sensor surfaces. It provides a reusable surface due to the reversible conjugation of ligand molecules on the NPs; this can pave the way for creating flexible, regenerable biosensor platforms for multi-use disease diagnostics for gold-surface-based sensors.

## Figures and Tables

**Table 1:** Characterization parameters of NPs.

Code	MW of PEG ^a^ (kDa)	Thiol Amount ^b^ (mM)	Diameter ^c^ (nm)	PDI (%)	Intensity (%)	Diameter ^d^ (nm)	Zeta Potential (mV)
NP1	2	-	177.5	17.28	100	137.6	−5.77 ± 6.97
NP2	6	-	89.60	10.23	90.1	114.7	−4.67 ± 7.92
NP3	10	-	144.1	19.37	95.6	101.8	−12.0 ± 5.53
NP4	2	1.350	224.5	7.13	100.0	137.1	−11.7 ± 6.79
NP5	6	1.615	121.3	11.89	97.6	116.8	−14.9 ± 3.38
NP6	10	0.434	151.6	14.87	98.5	122.2	−21.1 ± 4.25

^a^ Molecular weight of PEG polymer in PEG-diMA used to synthesize NPs. ^b^ Thiol amounts in NPs measured using Ellman’s assay. ^c^ Size values measured using DLS instrument in dH_2_O. ^d^ Size values measured via SEM under high vacuum.

**Table 2:** Characterization parameters of NP-coated surfaces.

No	Coating Material	MW of PEG ^a^ (kDa)	Water Contact Angle ^a^ (°)	Thickness ^b^ (nm)
1	none	-	24.07	-
2	NP1	2	9.75	378.3
3	NP2	6	20.35	122.1
4	NP3	10	12.42	189.3
5	NP4	2	26.28	283.3
6	NP5	6	18.67	84.9
7	NP6	10	12.64	116.4

^a^ Contact angle values measured by placing a 5 µL water droplet on the surface. ^b^ Thickness of NP layers on gold-coated glass surfaces measured via SEM. Maximum values are shown.

## Data Availability

All data will be made available upon acceptance and publication.

## Supplementary Materials

The following supporting information can be downloaded from https://www.mdpi.com/article/10.3390/biom15060767/s1. Figure S1: Zeta potential results of thiolated (NP 1–3) and nonthiolated (NP 4–6) NPs; Figure S2: SEM images of thiolated (NP 1–3) and nonthiolated (NP 4–6) NPs; Table S1: SEM results for prepared nanoparticles via EDT and EDS; Figure S3: Bare glass surface (left) and 100-nm-thick gold-coated glass surface (right); Table S2: Characterization details of linear PEG-diMA polymer-coated surfaces; Figure S4: SEM results for NP5 after incubation in water; Figure S5: HPLC traces for NTSR2-antibody solution in which the peptide-conjugated NP-coated gold surface was incubated, at different time points; Figure S6: HPLC traces for NTSR2-antibody solution.

## Abbreviations

The following abbreviations are used in this manuscript:

NP	Nanoparticle
DLS	Dynamic light scattering
DTT	Dithiothreitol
EDS	Energy-dispersive X-Ray spectroscopy
FT-IR	Fourier-transform infrared spectroscopy
HPLC	High-performance liquid chromatography
NTS	Neurotensin
NTSR	Neurotensin receptor
PEG	Poly(ethylene glycol)
PDS	Pyridyldisulfide
NMR	Nuclear magnetic resonance spectroscopy
MA	Methacrylate
