# Supplementary material for: Preparation of Nanoparticle-Immobilized Gold Surfaces for the Reversible Conjugation of Neurotensin Peptide
Source: Biomolecules. 2025 May 27;15(6):767. doi: 10.3390/biom15060767 (PMC12191028; doi:10.3390/biom15060767)
Supplement: Supplementary file 1 [file biomolecules-15-00767-s001.zip › biomolecules-3604799-supplementary.pdf]

Preparation of Nanoparticle Immobilized Gold Surfaces for the Reversible Conjugation of Neurotensin Peptide

Hidayet Gok[1], Deniz Gol[2], Betul Zehra Temur[3], Nureddin Turkan[4], Ozge Can[2,3,5], Ceyhun Ekrem Kirimli[2,5], Gokcen Ozgun[6] and Ozgul Gok[2,5,7,*]

[1] Department of Nanoscience and Nanoengineering, School of Engineering and Natural Sciences, Istanbul Medeniyet University, 34720 Istanbul, Turkey; hidayetgok76@gmail.com

[2] Department of Biomedical Engineering, Institute of Natural and Applied Sciences, Acibadem Mehmet Ali Aydinlar University, 34752 Istanbul, Turkey; denizgol1997@gmail.com (D.G.); ozge.can@acibadem.edu.tr (O.C.); ceyhun.kirimli@acibadem.edu.tr (C.E.K.)

[3] Department of Medical Biotechnology, Institute of Health Sciences, Acibadem Mehmet Ali Aydinlar University, 34752 Istanbul, Turkey; betulzkarakus@gmail.com

[4] Department of Physics Engineering, Faculty of Engineering and Natural Sciences, Istanbul Medeniyet University, 34720 Istanbul, Turkey; nureddin.turkan@medeniyet.edu.tr

[5] Department of Biomedical Engineering, Faculty of Engineering and Natural Sciences, Acibadem Mehmet Ali Aydinlar University, 34752 Istanbul, Turkey

[6] Department of Medical Biotechnology, Institute of Health Sciences, Acibadem University, 34752 Istanbul, Turkey; gokcen.ozgun@acibadem.edu.tr

[7] Department of Biomaterials, Institute of Natural and Applied Sciences, Acibadem Mehmet Ali Aydinlar University, 34752 Istanbul, Turkey

* Correspondence: ozgul.gok@acibadem.edu.tr; Tel.: +90-216-500-4188

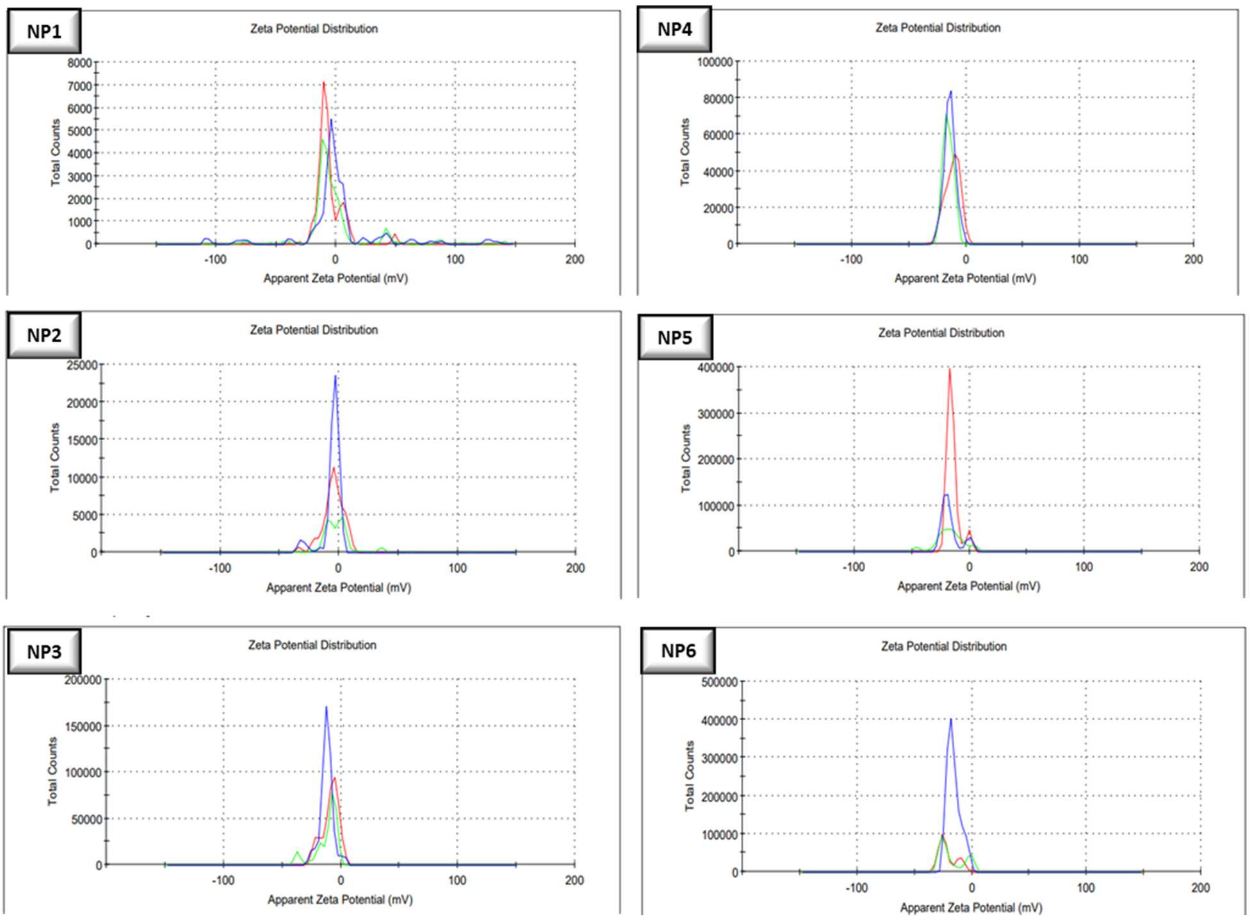

Figure S1. Zeta potential results of thiolated (NP 1–3) and nonthiolated (NP 4–6) NPs.

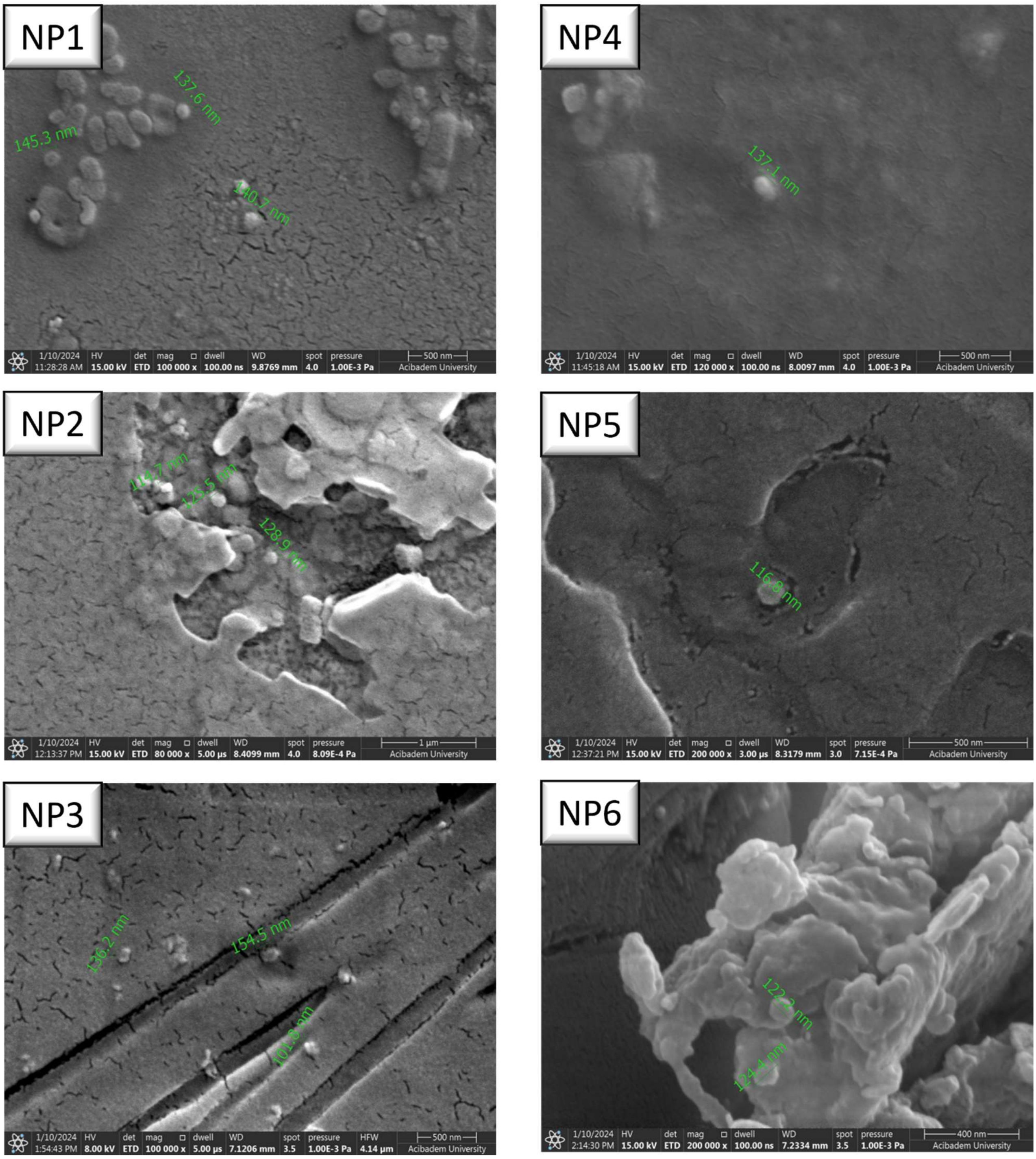

Figure S2. SEM images of thiolated (NP 1–3) and nonthiolated (NP 4–6) NPs.

Table S1. SEM results for prepared nanoparticles via EDT and EDS.

Code	MW of PEG (kDa)	Diameter (nm)	C (Weight %)	O (Weight %)	S (Weight %)
NP1	2	137,6	13,3	44	-
NP2	6	114,7	10,2	51,2	-
NP3	10	101,8	6,7	26,8	-
NP4	2	137,1	19,5	38,9	0,3
NP5	6	116,8	13,6	57,6	0,2
NP6	10	122,2	17,6	45,2	0,3

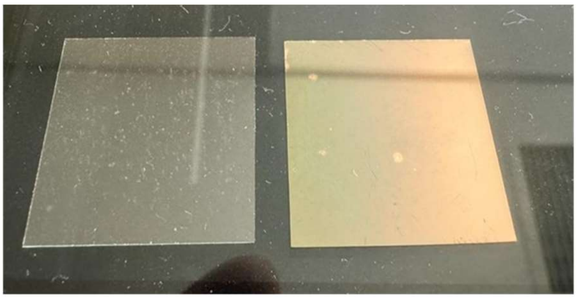

Figure S3. Bare glass surface (left) and 100-nm-thick gold-coated glass surface (right).

Table S2. Characterization details of linear PEG-diMA polymer-coated surfaces.

No	MW of PEGa (kDa)	Water Contact Angle (°)
1	2	16.81
2	6	12.46
3	10	11.10

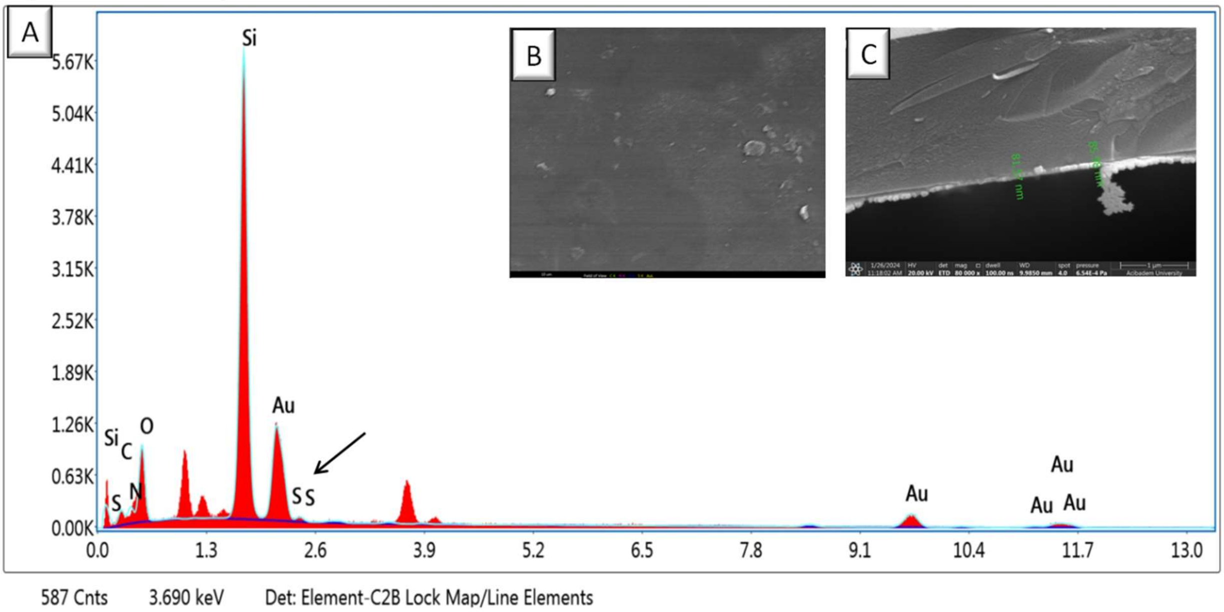

Figure S4. SEM results for NP5 after incubation in water.
(**a**) EDS spectrum and (**b**) Top-view EDS mapping, and (**c**) Side-view of the surface.

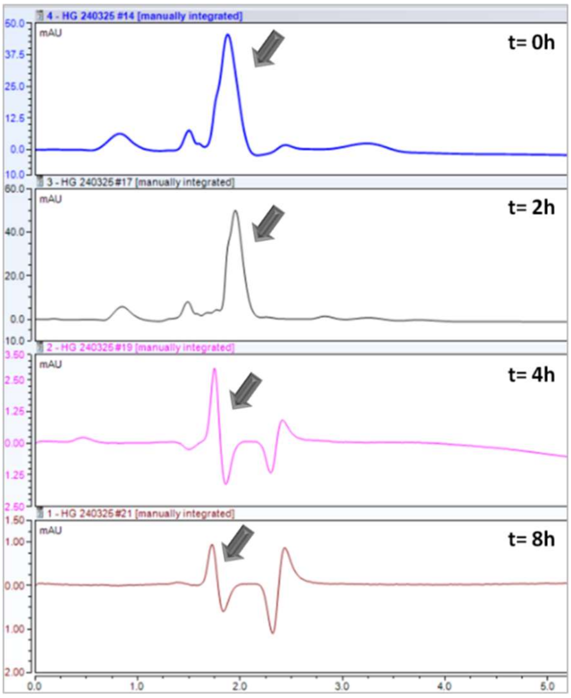

Figure S5. HPLC traces for NTSR2-antibody solution in which the peptide-conjugated NP-coated gold surface was incubated, at different time points.

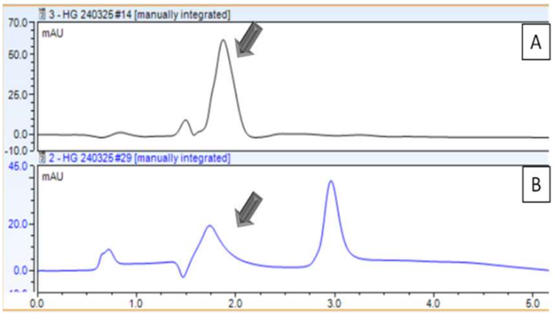

Figure S6. HPLC traces for NTSR2-antibody solution, (**a**) at the beginning of incubation for the binding experiment and (**b**) after its detachment in an acidic environment (pH 3.0).
